# Fully phase-stabilized quantum cascade laser frequency comb

**DOI:** 10.1038/s41467-019-10913-7

**Published:** 2019-07-03

**Authors:** Luigi Consolino, Malik Nafa, Francesco Cappelli, Katia Garrasi, Francesco P. Mezzapesa, Lianhe Li, A. Giles Davies, Edmund H. Linfield, Miriam S. Vitiello, Paolo De Natale, Saverio Bartalini

**Affiliations:** 1 CNR-Istituto Nazionale di Ottica and LENS, Via N. Carrara 1, 50019 Sesto Fiorentino, FI Italy; 2 NEST, CNR—Istituto Nanoscienze and Scuola Normale Superiore, Piazza S. Silvestro 12, 56127 Pisa, Italy; 3 School of Electronic and Electrical Engineering, University of Leeds, Leeds, LS2 9JT UK; 4 ppqSense Srl, Via Gattinella 20, 50013 Campi Bisenzio, FI Italy

**Keywords:** Mode-locked lasers, Quantum cascade lasers, Terahertz optics, Frequency combs

## Abstract

Miniaturized frequency comb sources across hard-to-access spectral regions, i.e. mid- and far-infrared, have long been sought. Four-wave-mixing based Quantum Cascade Laser combs (QCL-combs) are ideal candidates, in this respect, due to the unique possibility to tailor their spectral emission by proper nanoscale design of the quantum wells. We demonstrate full-phase-stabilization of a QCL-comb against the primary frequency standard, proving independent and simultaneous control of the two comb degrees of freedom (modes spacing and frequency offset) at a metrological level. Each emitted mode exhibits a sub-Hz relative frequency stability, while a correlation analysis on the modal phases confirms the high degree of coherence in the device emission, over different power-cycles and over different days. The achievement of fully controlled, phase-stabilized QCL-comb emitters proves that this technology is mature for metrological-grade uses, as well as for an increasing number of scientific and technological applications.

## Abstract (web summary)

Here, the authors demonstrate full stabilization and control of the two key parameters of a four-wavemixing-based quantum cascade laser comb with metrological precision. These fully-controlled, frequency scalable comb emitters will allow an increasing number of mid- and far-IR applications.

## Introduction

Optical frequency comb synthesizers (FCs)^1^ are laser sources covering a broad spectral range with a number of discrete, equally spaced and highly coherent frequency components, fully controlled through only two parameters: the frequency separation between adjacent modes and the carrier offset frequency. Providing a phase-coherent link between the optical and the microwave/radio-frequency regions^2^, FCs have become groundbreaking tools for precision measurements^3,4^. Despite these inherent advantages, developing miniaturized comb sources across the whole infrared (IR), with an independent and simultaneous control of the two comb degrees of freedom at a metrological level, has not been possible, so far. Recently, promising results have been obtained with innovative sources, namely diode-laser-pumped microresonators^5,6^ and quantum cascade lasers frequency combs (QCL-combs). In particular, the latter can benefit from a smaller footprint electrical pumping scheme, combined with an *ad-hoc* tailoring of the spectral emission in the 3–250 µm range, by quantum engineering^7^. However, due to the QCL-combs active region structure, the short gain recovery time of the laser prevents stable pulse train formation^8,9^ and, therefore, classical pulsed passive mode locking has not been achieved, yet. Pulsed emission has been demonstrated via active mode-locking^10,11^, with pulse duration, in the best cases, of few picoseconds. An alternative route to QCL-based FCs, able to achieve a much broader spectral coverage, is based on four-wave-mixing (FWM) parametric generation in QCLs^12,13^. These combs, whose emission profile is not pulsed, are based on multimode-emitting, broad-gain Fabry–Pérot QCL devices with low group velocity dispersion.

Thorough characterizations of FWM-based QCL-combs, recently performed both in the mid-IR^14,15^ and in the far-IR^15,16^, have demonstrated that FWM is a strong mode-locking mechanism, providing tight phase relation among the modes simultaneously emitted by the devices. However, while QCL-based FCs have been already used in a variety of sensing setups, mostly related to dual-comb spectroscopy^17,18^, their relevance as metrological-grade, phase-stabilized sources has not been proven, yet. In fact, simultaneous stabilization of the two key comb degrees of freedom requires two suitable and independent actuators. Although reliable procedures for stabilizing the QCL-comb modes spacing^12,19^ or the frequency of a single mode^20^ have been reported, independent control and tuning of carrier offset and spacing, together with an effective overall phase stabilization has represented, for a long time, a non-trivial breakthrough.

Here, we demonstrate full stabilization and control of the two key parameters of a FWM-based QCL-comb against the primary frequency standard. Our technique, here applied to a far-IR emitter and open ended to other spectral windows, enables Hz-level narrowing of the individual comb modes, and metrological-grade tuning of their individual frequencies, which are simultaneously measured with an accuracy of 2 × 10^−12^, limited by the frequency reference used. These fully-controlled, frequency-scalable comb emitters will allow an increasing number of mid- and far-IR applications, including quantum technologies, due to the quantum nature of the gain media^21^.

## Results

### Experimental setup

A detailed description of the QCL used in the present work can be found in Methods section. The bias-dependent emission bandwidth displays a maximum frequency coverage of 1.3 THz^22^, and is centered at 2.9 THz. The mode spacing, defined by the cavity length, can be electrically extracted through the measurement of the intermode beat-note (IBN—*f*_IBN_ ~ 17.45 GHz).

**Fig. 1:**
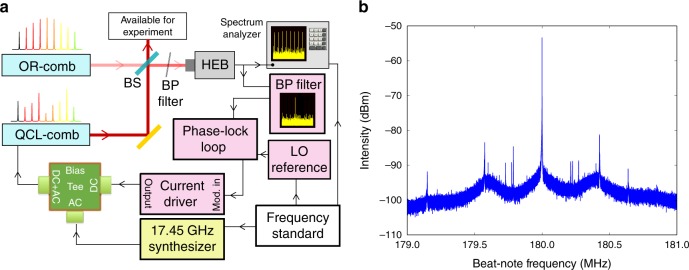
Experimental set-up. **a** Schematics of the experimental setup employed for full stabilization, control and characterization of the QCL-comb. The beams of the OR-comb and QCL-comb are superimposed by means of a beam splitter (BS) and then mixed on a fast detector (HEB: hot-electron bolometer). The HEB signal is acquired on a spectrum analyzer (Tektronics RSA5106A) while the two stabilization loops act on the QCL bias. The yellow background object represents the RF synthesizer used for injection locking, while the pink background objects are related to offset stabilization through phase-lock loop (BP filter: band-pass filter, LO reference: local oscillator reference). All the oscillators and the spectrum analyzer are frequency-referenced to the primary frequency standard. **b** Phase-locked beat-note signal acquired with a 2 MHz span and 10 Hz RBW. The two sidebands indicate a phase-lock electronic bandwidth of 400 kHz

The experimental setup used for full stabilization and characterization of the QCL-comb is shown in Fig. 1a, and it is based on multi-heterodyne dual-comb detection. An amplified mode-locked Erbium-doped fiber fs-laser (Menlo Systems, model FC1500) is optically rectified in a single-mode Lithium Niobate waveguide^23^, providing a zero-offset free-standing THz frequency comb^24,25^ (OR-comb). The repetition rate (*f*_rep_) of the pump laser can be tuned around 250 MHz, being the same for the generated THz OR-comb, while the average power of 350 mW at 1.55 μm generates an overall THz average power of about 14 μW. The QCL is mounted on the cold finger of a liquid Helium cryostat at a fixed heat sink temperature of 25 K, and it is driven in continuous-wave (CW) mode by an ultra-low noise current driver (ppqSense, QubeCL-P05) around 0.32 A. The OR-comb and the QCL-comb beams are overlapped by means of a wire grid polarizer (WGP) and generate the dual-comb multi-heterodyne signal on a fast non-linear detector, i.e. a hot electron bolometer (HEB—Scontel RS0.3-3T1). This frequency mixing results in a down-converted radio-frequency comb (RF-comb), which carries full information on the QCL-comb emission, and that allows its thorough characterization through Fourier-transform Analysis of Comb Emission (FACE) technique^15^.

A frequency synthesizer, oscillating at a frequency close to the QCL cavity round trip time (around 17.45 GHz) is used for injection locking the QCL-comb spacing, while a phase-locked loop (PL-loop) circuit, on board the current driver, is used to stabilize the frequency of one down-converted mode against a local oscillator frequency around 180 MHz. The phase-lock loop has a 400 kHz electronic bandwidth and is capable of effectively concentrating the power of the QCL-comb mode within the OR-comb mode width (see Fig. 1b). The two signals are coupled to the QCL by a cryogenic bias-tee (Marki Microwave, mod. BT-0024SMG) placed on chip, very close to the QCL device. The synthesizer, the PL-loop local oscillator and the spectrum analyzer used for the acquisitions are tightly ruled by a GPS-Rb-quartz frequency reference chain.

### Phase-locking the QCL-comb

**Fig. 2:**
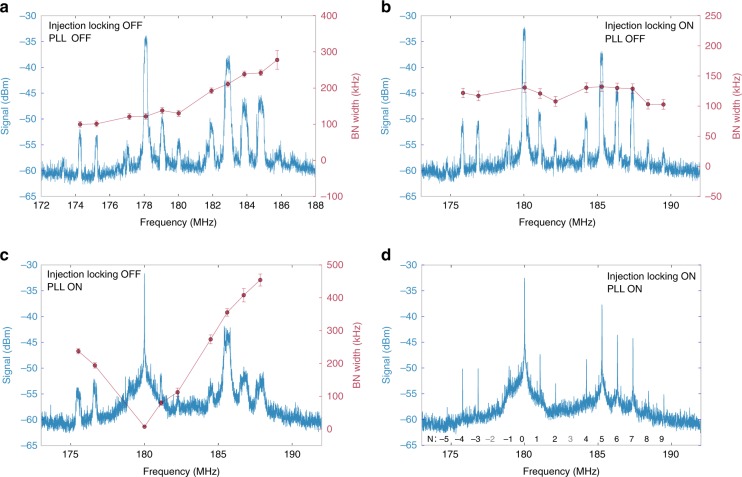
Radio frequency (RF) spectra. RF-comb spectra (blue traces) and related width of each beat-note (red dots), the uncertainty bars have been calculated as standard deviation on a set of 20 acquisitions. In all the acquisitions the optically-rectified THz frequency comb is fully stabilized, while the QCL-comb is: **a** free-running; **b** stabilized mode spacing and free-running offset frequency; **c** free-running spacing and stabilized offset; **d** fully stabilized. The order *N* of each comb mode is labeled in **d**, *N* = 0 indicating the mode used for offset frequency stabilization

Figure 2a shows the RF-comb spectrum of the free-running QCL-comb (blue trace) acquired by a real-time spectrum analyzer. The beat-notes (BNs) pattern distinctly retraces the QCL-comb Fourier transform infrared (FTIR) spectrum measured in air (Methods section). In these conditions, the emission linewidth of the OR-comb modes does not contribute to the BNs width, which shows a linear increase (about 14.4 kHz per mode) with the QCL-comb order *N*. This increment is close to the IBN width for a comparable acquisition time, confirming that the observed trend can be ascribed to frequency spacing fluctuations. In this scenario, a full active stabilization of the comb emission frequencies can be performed by means of two independent actuators.

On one hand, the mode spacing of the QCL-comb can be stabilized through injection locking (Fig. 2b), by feeding to the QCL a radio-frequency signal (*f*_RF_) approaching the QCL cavity round-trip frequency (f_IBN_). When injection locking is active, the QCL-comb frequency spacing fluctuations are drastically reduced (Fig. 2b), while only the mode-independent offset-frequency fluctuations survive, as confirmed by the roughly constant width (120 kHz) of the BN signals (red dots).

On the other hand, any mode of the RF-comb can be singly stabilized to a reference oscillator (*f*_LO_), using a phase-lock loop (PL-loop) acting on the QCL driving current (Fig. 2c) (see Methods). In this case, in absence of any QCL-comb mode spacing stabilization, the BN linewidths linearly increase with the order *N* of the comb tooth, with a slope of about 66.5 kHz/mode, much larger than that retrieved in free running conditions (Fig. 2a). Indeed, the offset fluctuations of the stabilized mode propagate to the adjacent modes as spacing fluctuations. Consequently, a significant broadening is observed for the BNs distant from the stabilized mode.

Finally, simultaneous implementation of both these processes results in a full stabilization of the QCL-comb (shown in Fig. 2d), as confirmed by the linewidth of the acquired BN signals, that are all resolution bandwidth (RBW) limited. It is worth noting that both the stabilization signals are sent to the QCL-comb as electrical signals, acting on its voltage/current, over two well separated frequency ranges: the RF range for the 17.45 GHz signal that locks the mode spacing, and the DC/low frequency range for the PL-loop, with a 400 kHz bandwidth. Actually, this configuration only requires a RF source for mode spacing stabilization, and one single-frequency metrological-grade THz signal^23,26,27^, falling into the QCL-comb emission spectrum, for offset frequency stabilization. To this purpose, in this work, we have used one mode of the OR-comb, while exploiting its complete emission for an exhaustive characterization of the QCL-comb.

### Frequency stability and comb parameters independent tuning

**Fig. 3:**
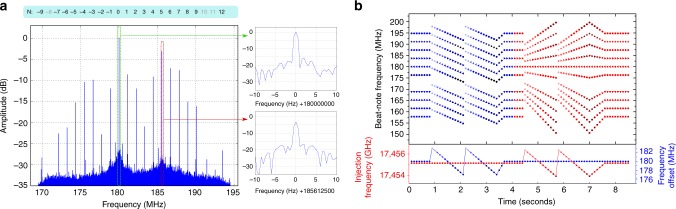
High-resolution comb spectra and frequency tuning. **a** RF-comb spectrum measured with 0.5 Hz RBW, limited by the acquisition time of 2 s. Insets: zoom on two RBW-limited peaks. **b** Fine tuning of the QCL-comb frequencies. First, the *f*_LO_ frequency of the PL-loop that stabilizes the comb-offset is tuned, in steps of 500 kHz, between 183 and 177 MHz. Then, the frequency of the injecting RF-signal (*f*_RF_) is tuned by 2.4 MHz, in steps of 200 kHz. Simultaneously, the RF-comb spectrum is acquired and the frequencies of individual modes are measured with a 100 Hz RBW

The QCL-comb mode frequencies can be simultaneously measured with high accuracy by acquiring RF spectra with a small RBW (0.5 Hz), limited by the spectrum analyzer maximum acquisition time of 2 s (Fig. 3a). The long acquisition time allows lowering the noise floor, thus enabling the detection of all the emitted modes. In the operating conditions presented in this work, the comb emission is made of 19 modes, with a total spectral coverage of about 400 GHz (−25-dB bandwidth). Moreover, the optical power of the individual modes can vary dramatically, which is another drawback of FWM based QCL-combs. Their RF frequencies coincide, within the RBW (Fig. 3a, insets), with the ones extrapolated from the reference signals frequencies (see Methods section). As a consequence, the emission linewidth of each QCL-comb mode is narrowed down to the linewidth of the OR-comb modes. This latter is ~2 Hz in 1 s, limited by the stability of the GPS-Rb-clock frequency reference used. The frequency reference accuracy of 2 × 10^−12^ also limits the measurement of the absolute QCL-comb THz frequencies, to a 6 Hz-level. However, for the most demanding applications, the GPS chain can be replaced with a fiber-delivered optical frequency standard, already available in our laboratories^28^, providing a stability of 1 × 10^−14^ in 1 s, and an accuracy of 2 × 10^−16^ ^29^.

By varying *f*_RF_ and *f*_LO_, a fine tuning of the corresponding QCL-comb parameters (spacing and offset, respectively) is obtained, as confirmed by the retrieved shifts of the RF-comb frequencies (Fig. 3b). Therefore, the double-locking procedure ensures tuning of the QCL-comb along two independent axes of its two-dimensional parameters space, which is a key requisite for full practical exploitation of this metrological-grade source. Actually, although the two actuators are not perfectly “orthogonal”, since the driving current affects both the spacing and offset frequencies, injection locking acts on the mode spacing only, allowing an independent tuning of the two parameters.

### Short and long term stability of the modal phases

**Fig. 4:**
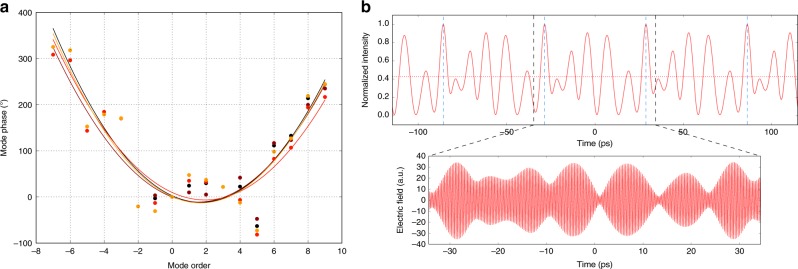
Measured phases. **a** Measured phases of each emitted mode. Different colors represent sets of measurements collected during different days and under different operating conditions. **b** QCL output intensity profile and electric field, reconstructed through one of the sets of phases shown in **a**

The employed FACE technique^15^ enables to retrieve the complete set of the modal phases, allowing to investigate their short-term noise and their long-term stability and reproducibility. To this purpose, several 2-seconds-long acquisitions of the multi-heterodyne signal have been analyzed. In order to assess the long-term reproducibility of the phases, different measurements were performed over different days, power cycling the QCL, changing the injection locking frequency and the device operating parameters (i.e. temperature and driving current). The device shows a quite stable phase pattern (Fig. 4a), with a phase reproducibility for individual modes always better than 50°. The retrieved phase relations show a parabolic trend, giving a 6.8(0.5) ps^2^/rad group delay dispersion (GDD). This value is close to that measured for a QCL-comb operating under comparable band alignment conditions, and without dispersion compensation geometry^15^. The retrieved phases and amplitudes of the QCL modes can also be used to reconstruct the QCL electric field and intensity emission profile, as shown in Fig. 4b. These reconstructions confirm that, while the QCL intensity is deeply modulated in time, it is far different from a short-pulse emission, since light is emitted during >40% of the round-trip time.

**Fig. 5:**
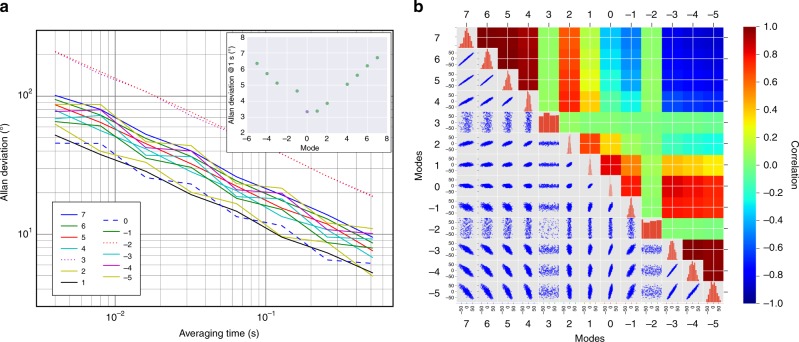
Phase noise analysis. **a** Allan variance analysis on the phase of each comb mode during a 2-seconds-long acquisition. The characteristic trends confirm that the measurements are affected by statistical noise. Inset: linear dependence of extrapolated Allan deviation at 1 s on the mode order. **b** Correlation analysis on the phases fluctuations. On the diagonal, the correlation matrix shows the distributions of each mode phase fluctuations. Under the diagonal, the mutual correlation scatterplots of the modal phases are presented, while the corresponding correlation coefficient, color coded, is presented in the upper part of the matrix. The correlation and anti-correlation among all modal phases (except for modes −2 and 3) confirms that the statistical noise detected is due to residual instabilities of the QCL-comb spacing

The short-term stability of the phases has been evaluated through calculation of the Allan deviations over a 2-seconds-long measurement for the modes labeled as −5… 7, sampled at 4 ms (Fig. 5a). Due to the poor S/N of modes −2 and 3 (see Fig. 3a), their phase cannot be determined at short timescales, leading to high values of the Allan deviation, dominated by instrumental noise. We can extrapolate the Allan deviations of the phases at ~1 s, as shown in Fig. 5a inset, and, except from these last two modes, they are all within 7°, which is a remarkable result if compared with the state-of-the-art in other spectral regions^30^.

Moreover, it is worth noting that the lowest noise level is measured on the phase-locked mode 0 and its neighbors, and progressively increases with the order *N*. This suggests that the dominant noise source is the residual noise from the spacing locking mechanism (i.e. injection locking), that coherently propagates along the comb. Such effect can be evidenced by calculating the correlation matrix among the modal phases (Fig. 5b). For modes −2 and 3, no correlation with the other modes appears, confirming that their fluctuations are limited by instrumental noise. At the same time, strong correlations and anti-correlations between modes which are, respectively, adjacent and opposite with respect to mode 0 can be seen. This behaviour can be found in traditional passively mode-locked frequency combs with residual phase noise and confirms the high level of coherence of the QCL-comb. Finally, principal components analysis can be performed on the correlation matrix by its diagonalization. The main eigenvalues normalized to the trace are (0.64, 0.29, 0.035, 0.025, 0.01…), confirming that the residual phase noise affecting the QCL-comb is, in fact, two-dimensional. The larger component is ascribed to comb modes spacing, rather than carrier offset frequency, suggesting that the injection locking mechanism should be improved.

### Phase noise power spectral density analysis

**Fig. 6:**
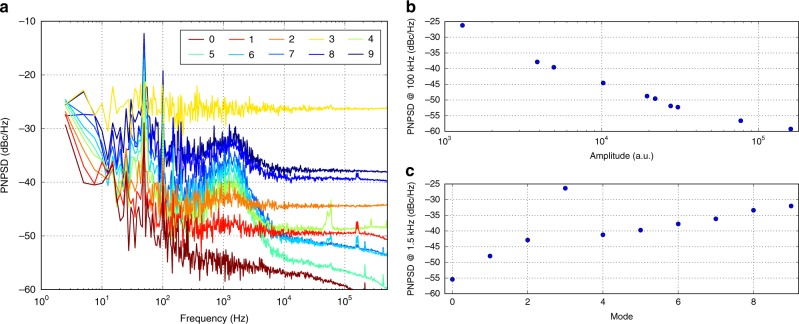
Phase Noise Power Spectral Density analysis. Simultaneous retrieval of the PNPSD of the beat-notes from the Fourier-transform spectra of the RF-comb in 2-second-long acquisitions (see Fig. 3a). **a** PNPSD of modes from 0 to 9, smoothed with an exponential law. **b** PNPSD level dependence on the amplitude of the modes at 100 kHz. **c** PNPSD dependence on the order of the modes at 1.5 kHz, corresponding to the excess noise peak

The simultaneous retrieval of the Phase Noise Power Spectral Density (PNPSD) of the beat-notes, available from the Fourier-transform spectra of the RF-comb in 2-second-long acquisitions (see Fig. 3a), allows the analysis of the phase noise at timescales shorter than 4 ms. Figure 6a shows the PNPSD of the modes from 0 to 9. The instrumental noise floor corresponds to the PNPSD flat levels above 10 kHz, that are proportional to the inverse of the amplitude of each considered beat-note (see Fig. 6b) and set the sensitivity limit of the phase-noise measurement. In fact, for the most intense modes (e.g. modes 0 and 5), a higher sensitivity is achieved (lower PNPSD). At frequencies below 10 kHz, the residual phase noise of the modes emerges from the noise floor. Apart from mode 3, with a S/N that is too small, in this range the PNPSD levels are proportional to the order of the modes, instead of being proportional to their amplitude, as shown in Fig. 6c. This confirms that the measured excess noise is related to the modes spacing, while its shape suggests a 2 kHz locking bandwidth for the injection locking mechanism. The PNPSD of modes from 0 to −5 is reported in Supplementary Fig. 2, and shows the same kind of behaviour. It is worth noting that the Allan deviation analysis presented in Fig. 5a is related to the portion of the PNPSD below 250 Hz, thus further confirming the proportional dependence on the modes order shown in Fig. 5a inset.

## Discussion

In conclusion, the reported results show that QCL-combs are well suited for the most demanding applications, achieving a Hz-level stabilization of each individual emitted mode, as well as a complete and independent control of the two key comb parameters. The retrieved phase relation, that enables reconstruction of the electric field and of the intensity profile, is impressively reproducible over different days, power cycles and operating conditions. The high degree of coherence induced by the FWM mode-locking mechanism is confirmed by a correlation analysis and PNPSD retrieval, showing a residual collective phase noise, due to an imperfect injection locking mechanism.

In the future, such a double-locking and phase characterization setup will be applied to dispersion-compensated devices, where comb operation at higher operating currents will ensure a broader spectral coverage. Novel comb-based setups will benefit from such fully phase-stabilized QCL-combs, addressing the most demanding metrological and sensing applications with miniaturized devices emitting in key spectral windows difficult to access, so far. Moreover, harnessing QCL nonlinearity, exploiting also novel phenomena, like *χ*^2^-cascaded frequency generation^31^, and making use of quantum simulation schemes in view of a fully-quantum design, QCL-combs with entangled modes and noise squeezed emission could become available, providing a solid-state miniaturized platform with a huge impact on a wide range of quantum technologies.

## Methods

### Design and fabrication of the QCL comb

The heterogeneous GaAs/AlGaAs heterostructure comprises three active modules, grown on a semi-insulating GaAs substrate by molecular beam epitaxy, exploiting alternating photon- and longitudinal optical (LO) phonon-assisted transitions between inter-miniband^22^. The number of periods, the order of the active region modules and the average doping were carefully arranged to give a flat gain and uniform power output across the whole spectrum. The gain medium of each modules is respectively centered at 3.5 THz, 3.0 THz, and 2.5 THz. The average doping was set to 3 × 10^16^ cm^−3^ ^32^. Laser bars were fabricated on a standard metal–metal processing that relies on Au–Au thermo-compression wafer bonding of the 17-μm-thick active region onto a highly doped GaAs substrate. Laser bars were defined using dry-etching, leading to almost vertical sidewalls narrow laser cavities without any unpumped regions, to favour stable continuous wave (CW) operation. A Cr/Au (8 µm/150 µm) top contact was then deposited on the top surface of the laser bars, leaving thin (5 µm) symmetrical side regions uncovered, to suppress undesired high order lateral modes. Thin (5-nm-thick) lateral nickel side absorbers were then deposited over the uncovered region using a combination of optical lithography and thermal evaporation. Laser bars 60–85 µm wide and 2 mm long were finally cleaved and mounted on a copper bar on the cold finger of a helium cryostat.

Supplementary Fig. 1a shows the current–voltage characteristics of the THz QCL-comb used in this work. The intermode beat-note retrieved at 320 mA, where the QCL shows a single narrow signal, is plotted in Supplementary Fig. 1b, while the corresponding in air Fourier transform infrared spectrum is graphed in panel 1c, for reference.

### Dual-comb down-conversion

In the dual-comb multi-heterodyne setup, the repetition rate *f*_rep_ of the OR-comb is tuned to be close to an integer submultiple of the frequency spacing *f*_s_ of the QCL modes, with a small detuning *f*_d_:

1 $$f_{\mathrm{s}} = k \cdot f_{{\mathrm{rep}}} + f_{\mathrm{d}}$$

In this configuration, the QCL modes at THz frequencies are down-converted to RF beat-note (BN) signals. In fact, considering the electrical field of the *N*^th^ mode of the QCL-comb:

2 $$E_{Q,N} = B_Ne^{i\left[ {2\pi \left( {f_{{\mathrm{off}}} + Nf_{\mathrm{s}}} \right)t + \varphi _N} \right]}$$

heterodyning it with the M^th^ mode of the OR-comb:

3 $$E_{O,M} = A_Me^{i\left[ {2\pi \left( {Mf_{{\mathrm{rep}}}} \right)t + \varphi _M} \right]}$$

being $M = k\cdot N$, the down-converted signal will carry the term:

4 $$A_MB_Ne^{i\left[ {2\pi \left( {f_{{\mathrm{off}}} + Nf_s - Mf_{{\mathrm{rep}}}} \right)t + \left( {\varphi _N - \varphi _M} \right)} \right]} = A_MB_Ne^{i\left[ {2\pi \left( {f_{{\mathrm{off}}} + Nf_d} \right)t + \left( {\varphi _N - \varphi _M} \right)} \right]}$$

In the above equations, *f*_off_ is the offset frequency of the QCL-comb (the OR-comb is offset-free), while *φ*_*N*_ and *φ*_*M*_ are the Fourier phases of the QCL-comb mode and of the OR-comb mode, respectively.

If *f*_d_ is chosen to be in the range of few MHz, the resulting BNs are RF signals, equally spaced by *f*_d_, that can be simultaneously analyzed by a spectrum analyzer. The signal in eq. 4 clearly bears information on both the amplitudes and phases of the two parent modes. In particular, the OR-comb phases are constant in time and their phase relation is linear, as for any mode-locked pulsed FC source^15^, while their amplitude is expected to be almost constant along the QCL-comb emitted spectrum^24^. Moreover, the OR-comb is naturally a zero-offset FC, whose modes can be fully stabilized at a metrological level by phase-locking its repetition rate to a 10 MHz quartz-clock disciplined by a Rb-GPS (global positioning system) clock with a stability of 6 × 10^−13^ in 1 s, and absolute accuracy of 2 × 10^−12^. On the other hand, the free-running QCL-comb emission frequencies will oscillate due to both offset and spacing fluctuations, preventing an accurate phase/frequency retrieval from the BN signals, especially for long observation times. Injection locking and phase locking to one OR-comb mode are used to remove these fluctuations, fully stabilizing the QCL-comb emission.

Moreover, since all the parameters governing the QCL-comb down conversion are referenced to the primary frequency standard, the frequency *f*_i_ of the ith RF-comb mode can be calculated with this same precision, simply considering that:

5 $$f_i = f_{{\mathrm{LO}}} + i\cdot \left( {kf_{{\mathrm{rep}}} - f_{{\mathrm{RF}}}} \right)$$

and these values can be compared to the ones experimentally retrieved with a 500 mHz accuracy, as reported in the main text.

This accuracy level allows also to simultaneously retrieve all the absolute THz frequencies of the QCL-comb modes with an accuracy limit set by the GPS-Rb-clock chain. In fact, if the RF beat-note at frequency *f*_i_ is originated from the *N*^th^ mode of the QCL comb, beating with the *M*^th^ mode of the OR-comb, one can write the relation:

6 $$f_{{\mathrm{QCL}},N} = M\cdot f_{{\mathrm{rep}}} \pm f_i$$

By tuning *f*_rep_ and leaving all the other parameters unaltered, it is possible to unambiguosly retrieve the order *M* (around 12,000), as thoroughly explained in ref. ^33^. Finally, while the accuracy on *f*_i_ is 500 mHz, the phase-locked *f*_rep_ follows the GPS-Rb-quartz chain accuracy, that provides the limiting uncertainty factor in the *f*_QCL*,N*_ measurement of about 6 Hz.

## Supplementary information

Supplementary Information

## Data Availability

The data supporting the findings of this study are available from the corresponding author upon reasonable request.
