# Supplementary material for: Fully phase-stabilized quantum cascade laser frequency comb
Source: Nat Commun. 2019 Jul 3;10:2938. doi: 10.1038/s41467-019-10913-7 (PMC6610094; doi:10.1038/s41467-019-10913-7)
Supplement: Supplementary file 1 — Supplementary Information [file 41467_2019_10913_MOESM1_ESM.pdf]

Supplementary Information

Fully phase-stabilized quantum cascade laser frequency comb

Consolino *et al*

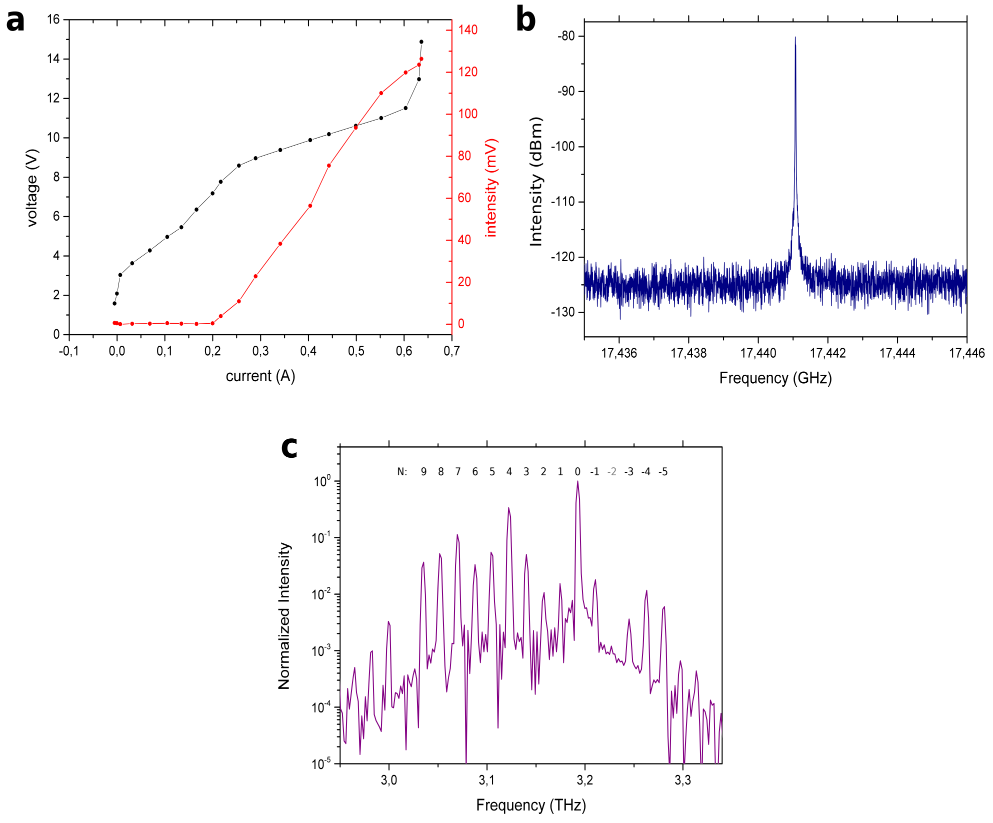

Supplementary Figure 1 Electrical and optical characterization of the QCL-comb. **a** current-voltage characteristics of the THz QCL-comb. **b** intermode beat-note (IBN) signal electrically retrieved. The IBN is 20 kHz wide at 20 ms integration time. **c** Fourier transform infrared spectrum measured at a heat sink temperature of 25 K and with QCL operating in continuous wave with a current of 0.32 A. The spectrum is acquired in rapid scan mode, in air, with a resolution of 0.125 cm -1 . The order *N* of each mode is labeled on the graph.

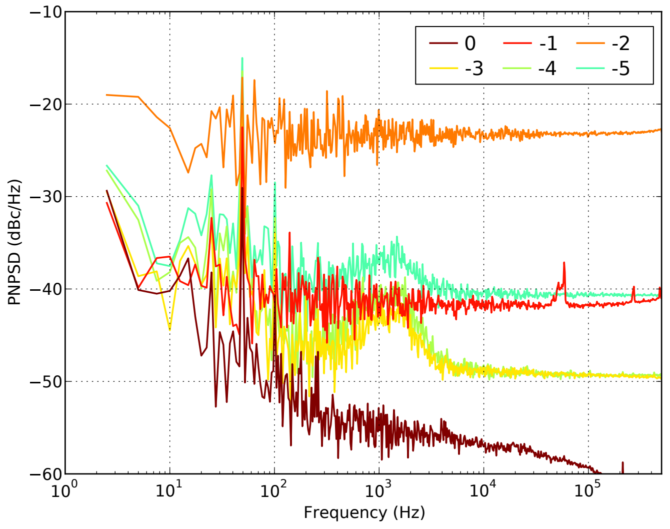

Supplementary Figure 2 Phase Noise Power Spectral Density analysis. Simultaneous retrieval of the PNPSD of the beat-notes from the Fourier-transform spectra of the RF-comb in 2-second-long acquisitions (see fig. 3a), for modes from 0 to -5.
